# Heat transfer of generalized second grade fluid with MHD, radiation and exponential heating using Caputo–Fabrizio fractional derivatives approach

**DOI:** 10.1038/s41598-022-22665-4

**Published:** 2023-03-30

**Authors:** Sehra Sehra, Afshan Noor, Sami Ul Haq, Saeed Ullah Jan, Ilyas Khan, Abdullah Mohamed

**Affiliations:** 1 Shaheed Benazir Bhutto Women University Peshawar, Peshawar, 25000 Khyber Pakhtunkhwa Pakistan; 2 Department of Mathematics, Islamia College Peshawar, Peshawar, 25000 Khyber Pakhtunkhwa Pakistan; 3 Department of Mathematics, College of Science Al-Zulfi, Majmaah University, Al-Majmaah, 11952 Saudi Arabia; 4 Research Centre, Future University in Egypt, New Cairo, 11835 Egypt

**Keywords:** Mathematics and computing, Physics

## Abstract

The aim of the present work is to apply the Caputo–Fabrizio fractional derivative to the heat transformation of unsteady incompressible second grade fluid. The effects of magneto hydro dynamic and radiation are analyzed. In governing equation of heat transfer nonlinear radiative heat is examined. Exponential heating phenomena is considered at boundary. Firstly, the dimensional governing equations with the initial & boundary conditions are converted into non-dimensional form. Exact analytical solutions are obtained for dimensionless fractional governing equations which consist of momentum and energy equations by using Laplace transform method. Special cases are investigated of the obtained solutions and it is noticed that some well-known results are achieved published in literature from these special cases. At the end, for graphical illustration the influences of different physical parameters like radiation, Prandtl, fractional parameter, Grashof numbers and Magneto hydro dynamic are checked graphically.

## Introduction

The theory of derivatives with fractional order has great importance in daily life. As integer order, the theory of non-integer order is also oldest. It is the branch of mathematics, a few years ago this concept was limited only in mathematics, but now a days the principles of fractional calculus have been often carried out to different fields such as fluid dynamics, bio engineering, electromagnetism, fluid mechanics, finance, electrochemistry, viscoelasticity, in biology the model of neurons, applied mathematics^1^. In fluid dynamics the non-integer derivative concept have been used to investigate viscoelastic process like polymers in the glassy state and glassy transition^2^. A few years ago, fractional order derivatives have been seen is an effective tool from which a suitable physical concepts generalization can be gained. There are so many other definitions of derivatives with non-integer order but the Caputo fractional and Riemann-Liouvilli fractional derivatives are used in different real world phenomena’s^3,4^. Everyone knows that such methods show difficulties in application. Such as the derivative of a constant is non-zero in Riemann-Liouvilli fractional order derivative and also it has singular kernel. These difficulties are removed by Caputo and gave the concept in which the constant has zero derivative but have still singular kernel. After all these, Fabrizio & Caputo presented the idea of non-integer order derivative in which constant has derivative zero & without singular kernel. By Laplace technique Caputo-Febrizio fractional derivative is easy to find exact solution. Many existing fluid models have been examined and fractional order derivative has been developed. Some of well-known fluid models are presented here like, the Oldroyd-B, Maxwell, grade second, Burger and Jeffery fluid models etc. The Burger, Maxwell and Oldroyd models are rate type models, while the grade second are of differential type^5^. According to Tan et al.^6^ investigated the generalized unsteady flow of grade second non-Newtonian fluid between two parallel plates with the model of non- integer derivatives. Recently, Friedrich^7^, examined the fluid model of ordinary Maxwell fluid with fractional order derivative generalized the function of relaxation and retardation. In the earlier studies, Tan et al.^8^ analyzed a short note on non-integer Maxwell fluid with flow of unsteady viscoelastic fluid between two parallel plates. The non-integer viscoelastic Maxwell fluid model with one directional periodic fluid flow studied in^9^. The model of fractional Maxwell fluid of viscoelastic in pipe was examined by Yin et al.^10^. Brikman type fluid by Caputo fractional derivative is investigated in^11^. The effects of parameters in generalized second grade fluid is discussed in^12^. The Maxwell non-integer order derivative for the Stokes first problem is studied in^13^. Khan et al.^14^ studied the generalized modified Darcy’s law with Oldroyd-B fluid to obtain exact solutions for Magnetohydrodynamic. Khan et al.^15^ studied Burgers fluid model of viscoelastic non-integer on accelerated flows. By using Caputo Fabrizio non-integer derivative studied heat transfer fluid of second grade over and oscillating perpendicular surface examined in^16^. Heat mass transfer investigated in the third grade fluid with chemical reaction upon a stretchable sheet fixed in a porous medium. Abbas et al.^17^ investigated the third grade fluid thermal diffusion with Darcy–Forchheimer relation upon a stretchable sheet. Analysis of heat transfer in Atangana–Baleanu derivative to Newtonian heating and convection flows of Caputo–Fabrizio with second grade fluids is investigated in^18^. Recently, by using derivative of Caputo Fabrizio non-integer and examined the exponential heating and Magnetohydrodynamic flow of second grade fluid in^19^. Saqib et al.^20^ studied Jeffery fluid flow by using Caputo–Fabrizio derivative and obtain exact solutions. Raptis et al.^21^ investigated MHD influence of thermal radiation over a stretchable sheet influence of heat radiation on MHD is studied in^22^. Purpose of this article is to discuss analysis of generalized second grade non-newtonian fluid on magneto hydrodynamic and heat radiation using Caputo–Fabrizio fractional derivative approach. On thermal aspect, exponential heating phenomena to be adopted.

## Mathematical formulation of the problem

**Figure 1:**
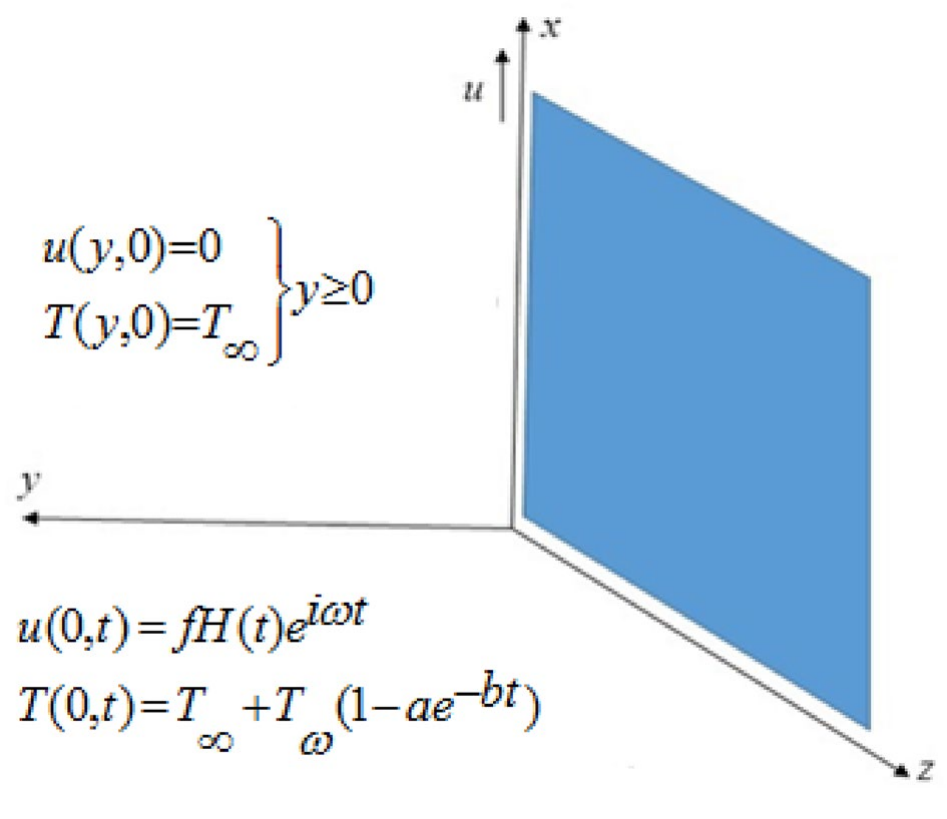
Geometry of the problem.

Consider the incompressible non-Newtonian second grade fluid. Initially for time t = 0 the temperature T∞ and velocity is zero. As time start for t = 0^+^, the fluid velocity becomes $fH(t)e^{i\omega t}$, here H(t) is the unit step function and temperature reaches $T_{\infty } + T_{\omega } (1 - ae^{ - bt} )$. According to all these assumptions temperature and velocity both are the function of space variable “y” and time “t” only. Now by usual Boussinesq’s approximation^16^, the unsteady flow is governed by the following set of partial differential equations. The schematic diagram used in fluid flow problem is represented geometrically by Fig. 1.

1 $$ \frac{\partial u(y,t)}{{\partial t}} = \nu \frac{{\partial^{2} u(y,t)}}{{\partial y^{2} }} + \frac{{\alpha_{1} }}{\rho }\frac{{\partial^{3} u(y,t)}}{{\partial y^{2} \partial t}} - \frac{{\sigma B_{0}^{2} u(y,t)}}{\rho } + g\beta_{T} (T(y,t) - T_{\infty } );\quad y,t > 0, $$

2 $$ \frac{\partial T(y,t)}{{\partial t}} = \frac{k}{{\rho C_{p} }}\frac{{\partial^{2} T(y,t)}}{{\partial y^{2} }} - \frac{1}{{\rho C_{p} }}\frac{{\partial q_{r} }}{\partial y};\quad y,t > 0, $$

For radiation approximation of Rosseland is used^23^, we have

$$ q_{r} = - \frac{{4\sigma^{*} }}{{3k^{*} }}\frac{{\partial T^{4} }}{\partial y}. $$

By neglecting the higher terms with the help of Taylor series we express T^4^ as a linear function,

$$ T^{4} \cong 4T_{\infty }^{3} T - 3T_{\infty }^{4} $$

Initial and boundary condition:

3 $$ u(y,0) = 0,T(y,0) = T_{\infty } ,\quad y > 0 $$

4 $$ u(0,t) = fH(t)e^{i\omega t} ,T(0,t) = T_{\infty } + T_{\omega } (1 - ae^{ - bt} ),\quad f \ne 0\;and\;t \ge 0, $$

5 $$ As\quad y \to \infty ,\quad t > 0\quad then\quad u(y,t) = T_{\infty } $$

Dimensionless Variable:

$$ y^{*} = \frac{fy}{\nu },t^{*} = \frac{{f^{2} t}}{\nu },\quad u^{*} = \frac{u}{f},\quad \theta^{*} = \frac{{T - T_{\infty } }}{{T_{\omega } }},\quad \alpha_{2} = \frac{{\alpha_{1} f^{2} }}{\mu \nu },\quad Gr = \frac{{\nu g\beta_{T} T_{\omega } }}{{f^{3} }},\quad M = \frac{{\sigma \nu B_{0}^{2} }}{{\rho f^{2} }}, $$

6 $$ N = \frac{{4\sigma^{*} T_{\infty }^{3} }}{{kk^{*} }}, \, \xi = 1 + \frac{4}{3}N, $$

After dimensionless Eqs. (1)–(5), we get

7 $$ \frac{\partial u(y,t)}{{\partial t}} = \frac{{\partial^{2} u(y,t)}}{{\partial y^{2} }} + \alpha_{2} \frac{{\partial^{3} u(y,t)}}{{\partial y^{2} \partial t}} - Mu(y,t) + Gr\theta (y,t), $$

8 $$ \Pr \frac{\partial \theta (y,t)}{{\partial t}} = \left( {1 + \frac{4}{3}N} \right)\frac{{\partial^{2} \theta (y,t)}}{{\partial y^{2} }}, $$

9 $$ u(y,0) = 0,\quad \theta (y,0) = 0,\quad y \ge 0 $$

10 $$ u(0,t) = H(t)e^{i\omega t} ,\quad \theta (0,t) = 1 - ae^{ - bt} ,\quad t \ge 0, $$

11 $$ As \, \quad y \to \infty ,\quad t \ge 0 \, \quad then\quad \, u(y,t) \to 0,\quad \theta \left( {y,t} \right) \to 0. $$

Now using the Caputo–Fabrizio time derivative in Eqs. (7) and (8) we get the system given below:

12 $$ D_{t}^{\alpha } u(y,t) = \frac{{\partial^{2} u(y,t)}}{{\partial y^{2} }} + \alpha_{2} D_{t}^{\alpha } \frac{{\partial^{2} u(y,t)}}{{\partial y^{2} }} - Mu(y,t) + Gr\theta (y,t), $$

13 $$ \Pr D_{t}^{\alpha } \theta \left( {y,t} \right) = \left( {1 + \frac{4}{3}N} \right)\frac{{\partial^{2} \theta (y,t)}}{{\partial y^{2} }}, $$

14 $$ D_{t}^{\alpha } u(y,t) = \frac{1}{1 - \alpha }\int_{0}^{t} {\exp \left( { - \frac{\alpha (t - \tau )}{{1 - \alpha }}} \right)u^{^{\prime}} (\tau )d\tau , \, \quad {\text{where }}\alpha \in (0,1).} $$

### Temperature computation

To obtain the solutions of the governing equations we use the Laplace transform technique. First we find the solution of energy equations because momentum equation depends on it. Now taking the Laplace transform of Eq. (12) with initial and boundary conditions Eqs. (9) and (10), we have the following equations:

15 $$ \frac{\Pr \gamma p}{{\xi (p + \alpha \gamma )}}\overline{\theta }(y,p) = \frac{{\partial^{2} \overline{\theta }(y,p)}}{{\partial y^{2} }}, $$

16 $$ \overline{\theta }(0,p) = \frac{1}{p} - \frac{a}{p + b},\quad \overline{\theta }(y,p) \to 0\quad as\quad y \to \infty . $$

Solving Eq. (15) with help of Eq. (16) we a transformed solution which is given

17 $$ \overline{\theta }(y,p) = \frac{1}{p}\exp \left( { - y\sqrt {\frac{\Pr \gamma p}{{\xi (p + \alpha \gamma )}}} } \right) - \frac{a}{p + b}\exp \left( { - y\sqrt {\frac{\Pr \gamma p}{{\xi (p + \alpha \gamma )}}} } \right), $$

Now to find the exact analytical solution of energy equation we take the inverse Laplace transform of Eq. (17) by using the Appendices A1 and A2, we obtain the solution which is presented in Eq. (18)

18 $$ \theta (y,t) = (1 - a)\varphi \left( {y,t;\frac{\Pr \gamma }{\xi },\alpha \gamma } \right) + a\psi \left( {y,t;\frac{\Pr \gamma }{\xi },\alpha \gamma , - b} \right),\quad 1 > \alpha > 0, $$

### Computations for velocity equation

To find the velocity equation we take the Laplace Transform of Eq. (12) with initial boundary conditions Eqs. (9) and (10), now the transformed equation with transformed initial boundary conditions are given in Eqs. (19) and (20):

19 $$ \frac{\gamma p}{{p + \alpha \gamma }}\overline{u}(y,p) = \frac{{\partial^{2} \overline{u}(y,p)}}{{\partial y^{2} }} + \alpha_{2} \frac{\gamma p}{{p + \alpha \gamma }}\frac{{\partial^{2} \overline{u}(y,p)}}{{\partial y^{2} }} - M\overline{u}(y,p) + Gr\overline{\theta }(y,p), $$

20 $$ \overline{u}(y,p) = \frac{1}{p - i\omega },\quad \overline{u}(y,p) \to 0\quad as\quad y \to \infty . $$

Solving Eq. (19) with help of Eq. (20) we get the transformed which is presented in Eq. (21)

$$ \overline{u}(y,p) = \frac{1}{p - i\omega }\exp \left( { - y\sqrt {\frac{{a_{1} p + a_{2} }}{{p + a_{3} }}} } \right) - \delta_{1} \frac{1}{p}\exp \left( { - y\sqrt {\frac{{a_{1} p + a_{2} }}{{p + a_{3} }}} } \right) + \delta_{2} \frac{1}{p + b}\exp \left( { - y\sqrt {\frac{{a_{1} p + a_{2} }}{{p + a_{3} }}} } \right) $$

$$ + \delta_{3} \frac{1}{{p + f_{1} }}\exp \left( { - y\sqrt {\frac{{a_{1} p + a_{2} }}{{p + a_{3} }}} } \right) + \delta_{4} \frac{1}{{p + f_{2} }}\exp \left( { - y\sqrt {\frac{{a_{1} p + a_{2} }}{{p + a_{3} }}} } \right) + \delta_{1} \frac{1}{p}\exp \left( { - y\sqrt {\frac{\Pr \gamma p}{{\xi (p + \alpha \gamma )}}} } \right) $$

21 $$ - \delta_{2} \frac{1}{p + b}\exp \left( { - y\sqrt {\frac{\Pr \gamma p}{{\xi (p + \alpha \gamma )}}} } \right) - \delta_{3} \frac{1}{{p + f_{1} }}\exp \left( { - y\sqrt {\frac{\Pr \gamma p}{{\xi (p + \alpha \gamma )}}} } \right) - \delta_{4} \frac{1}{{p + f_{2} }}\exp \left( { - y\sqrt {\frac{\Pr \gamma p}{{\xi (p + \alpha \gamma )}}} } \right), $$

where; $$a_{1} = \frac{M + \gamma }{{1 + \alpha_{2} \gamma }},a_{2} = \frac{M\alpha \gamma }{{1 + \alpha_{2} \gamma }},a_{3} = \frac{\alpha \gamma }{{1 + \alpha_{2} \gamma }},h_{1} = - \frac{Gr\xi }{{\Pr \gamma + \Pr \gamma^{2} \alpha_{2} - M\xi - \gamma \xi }},$$

$$ h_{2} = \frac{{Pr\alpha \gamma^{2} - 2M\xi \alpha \gamma - \gamma^{2} \alpha \xi }}{{\Pr \gamma + \Pr \gamma^{2} \alpha_{2} - M\xi - \gamma \xi }},h_{3} = \frac{{M\xi \alpha^{2} \gamma^{2} }}{{\Pr \gamma + \Pr \gamma^{2} \alpha_{2} - M\xi - \gamma \xi }}, $$

$$ f_{1} = \frac{{h_{2} }}{2} + \sqrt {\left( {\frac{{h_{2} }}{2}} \right)^{2} + h_{3} } ,f_{2} = \frac{{h_{2} }}{2} - \sqrt {\left( {\frac{{h_{2} }}{2}} \right)^{2} + h_{3} } ,\delta_{1} = \frac{{\alpha^{2} \gamma^{2} h_{1} }}{{f_{1} f_{2} }},\delta_{2} = \frac{{ah_{1} (\alpha \gamma - b)^{2} }}{{( - b + f_{1} )( - b + f_{2} )}}, $$

$$ \delta_{3} = \frac{{h_{1} ( - f_{1} + \alpha \gamma )^{2} }}{{f_{1} (f_{2} - f_{1} )}} + \frac{{ah_{1} ( - f_{1} + \alpha \gamma )^{2} }}{{( - f_{1} + b)( - f_{1} + f_{2} )}},\delta_{4} = \frac{{h_{1} ( - f_{2} + \alpha \gamma )^{2} }}{{f_{2} (f_{1} - f_{2} )}} + \frac{{ah_{1} ( - f_{2} + \alpha \gamma )^{2} }}{{( - f_{2} + b)( - f_{2} + f_{1} )}}. $$

To obtain the exact analytical solution of momentum equation we take the inverse Laplace transform of Eq. (21) we get the solution which is given in Eq. (22) by using the Appendices A1, A2 and A4,

$$ u(y,t) = \left( {e^{i\omega t} - \delta_{1} + \delta_{2} e^{ - bt} + \delta_{3} e^{{ - f_{1} t}} + \delta_{4} e^{{ - f_{2} t}} } \right) * h\left( t \right) + \delta \varphi \left( {y,t;\frac{\Pr \gamma }{\xi },\alpha \gamma } \right) $$

22 $$ - \delta_{2} \psi \left( {y,t;\frac{\Pr \gamma }{\xi },\alpha \gamma , - b} \right) - \delta_{3} \psi \left( {y,t;\frac{\Pr \gamma }{\xi },\alpha \gamma , - f_{1} } \right) - \delta_{4} \psi \left( {y,t;\frac{\Pr \gamma }{\xi },\alpha \gamma , - f_{2} } \right) $$

### Special cases

(i) In the absence of radiation effect $N = 0$ and neglecting the exponential heating of the plate.

In Eq. (8), when we put $N = 0$, then we obtain the solution in the form as given under:

$$ T\left( {y,t} \right) = \varphi \left( {y, \, t,{\text{ pr, }}\gamma , \, \alpha \gamma } \right) $$

where $\varphi \left( {y, \, t, \, p_{r} \gamma , \, \alpha \gamma } \right)$ is defined by the Appendix (A1).

The result is uniform to that in the published literature achieved by Shah and Khan^16^.

Also neglecting the radiation effect in Eq. (7) we get the solution for velocity equation which is given as under:

$$ u\left( {y,t} \right) = \psi \left( {y, t,a_{1} ,a_{2} ,i\omega } \right) = - \omega \int\limits_{0}^{t} {\sin \left[ {\omega \left( {t - \tau } \right)} \right]\varphi \left( {y, \tau ,a_{1} ,a_{2} } \right){\text{d}}\tau + i} \omega \int\limits_{0}^{t} {\cos \left[ {\omega \left( {t - \tau } \right)} \right]\varphi \left( {y, \tau ,a_{1} ,a_{2} } \right){\text{d}}\tau } $$

where $a_{1} = \frac{\gamma }{{1 + \alpha_{2} \gamma }}, \, a_{2} = \, \alpha a_{1}$ and $\varphi \left( {y, \tau ,a_{1} ,a_{2} } \right)$ is defined by the Appendix (A1).

The result is uniform to the one in published literature achieved by Shah and Khan^16^.

## Numerical results and discussions

**Figure 2:**
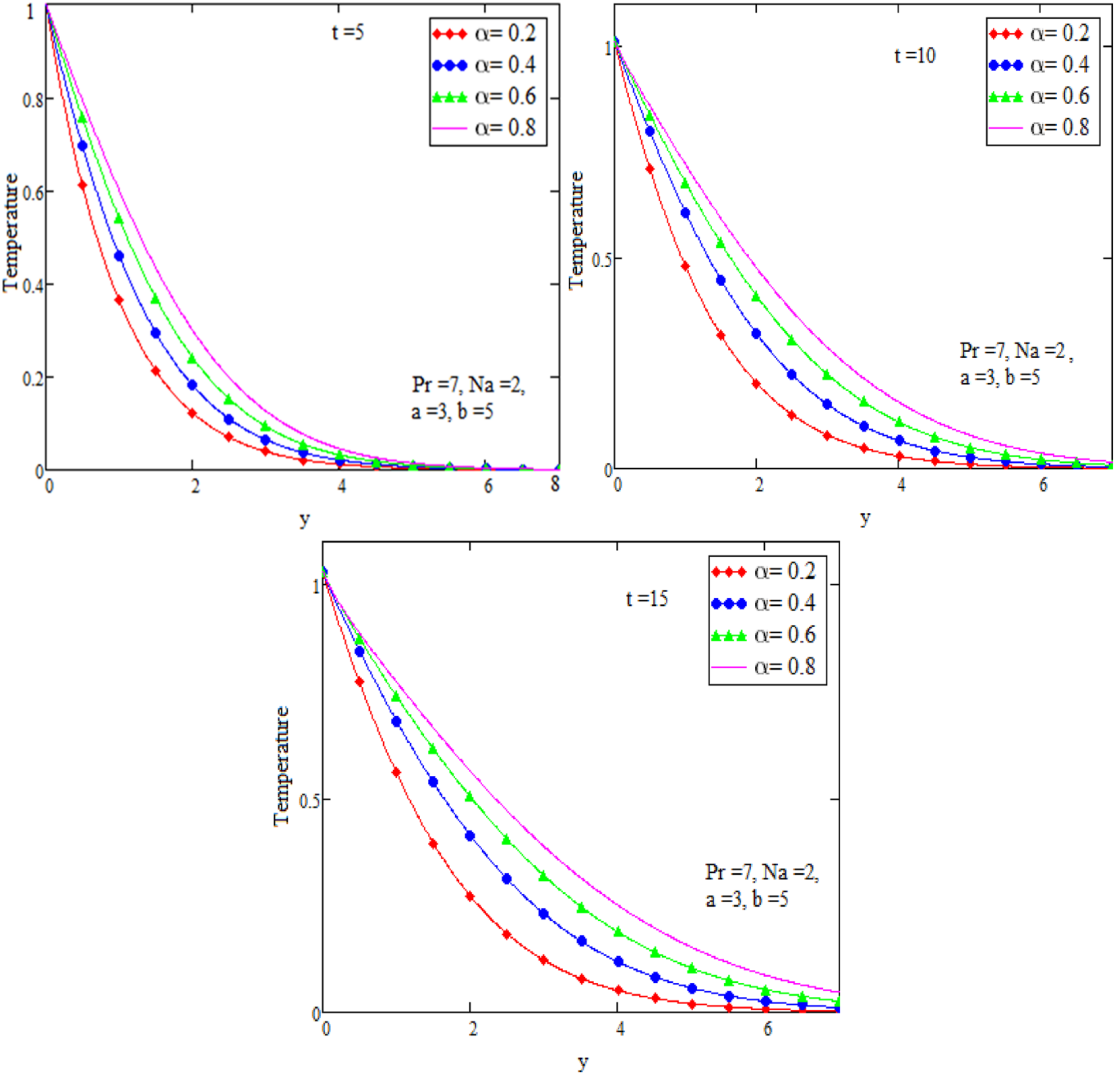
Graph of temperature for different value of alpha α.

**Figure 3:**
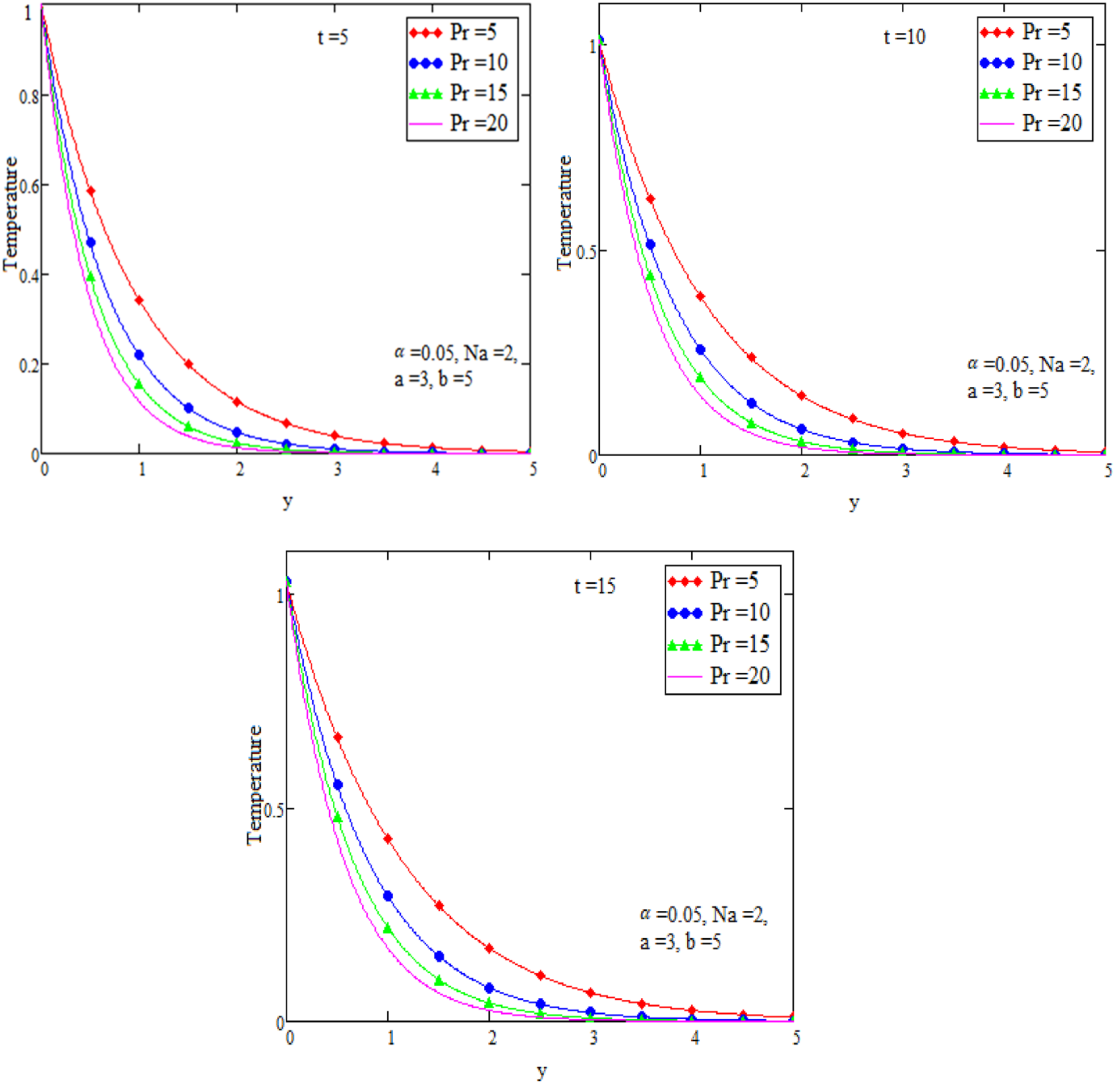
Graph of temperature for different value of Prandtl number Pr.

**Figure 4:**
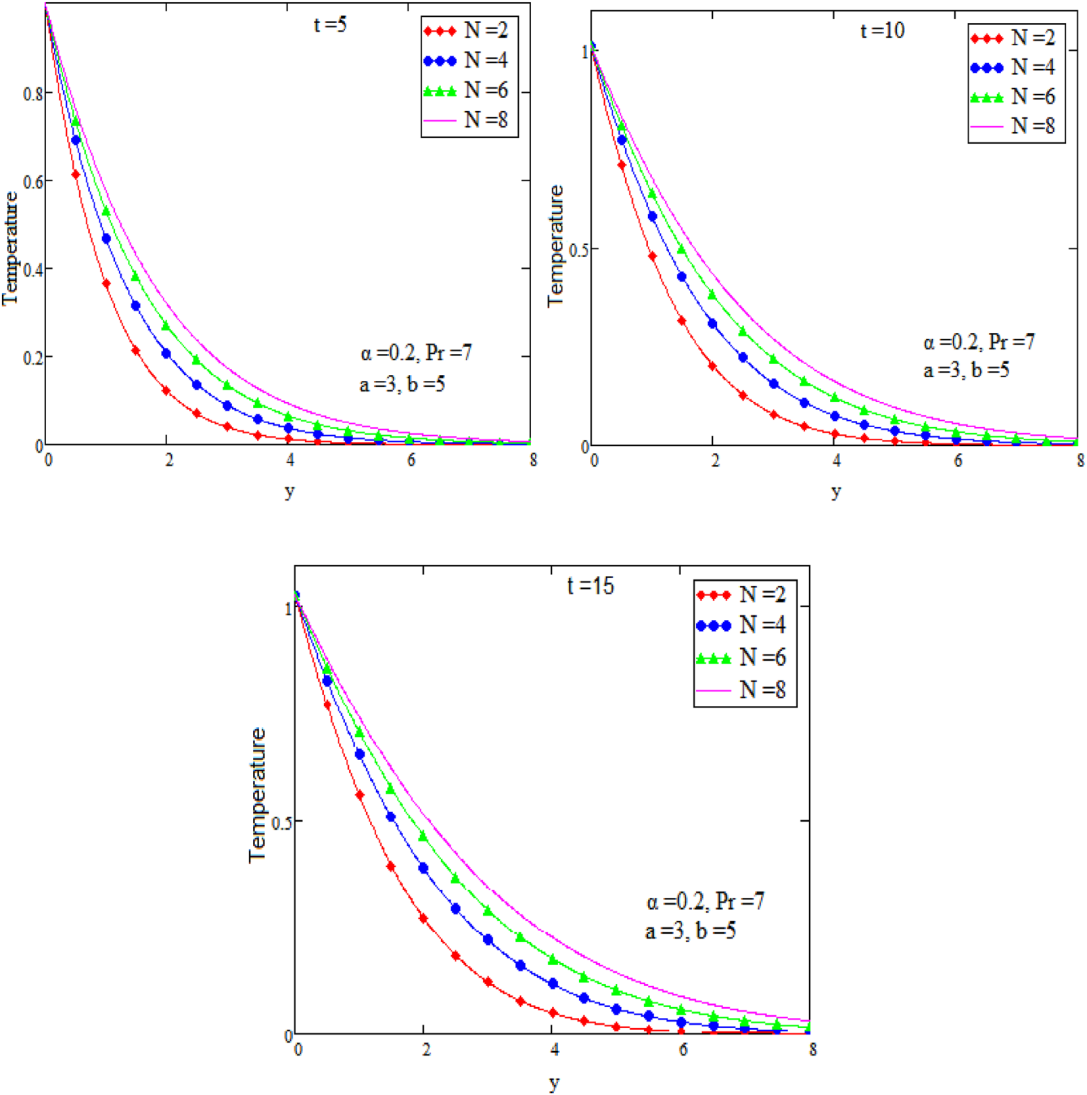
Graph of temperature for different value of radiation N.

**Figure 5:**
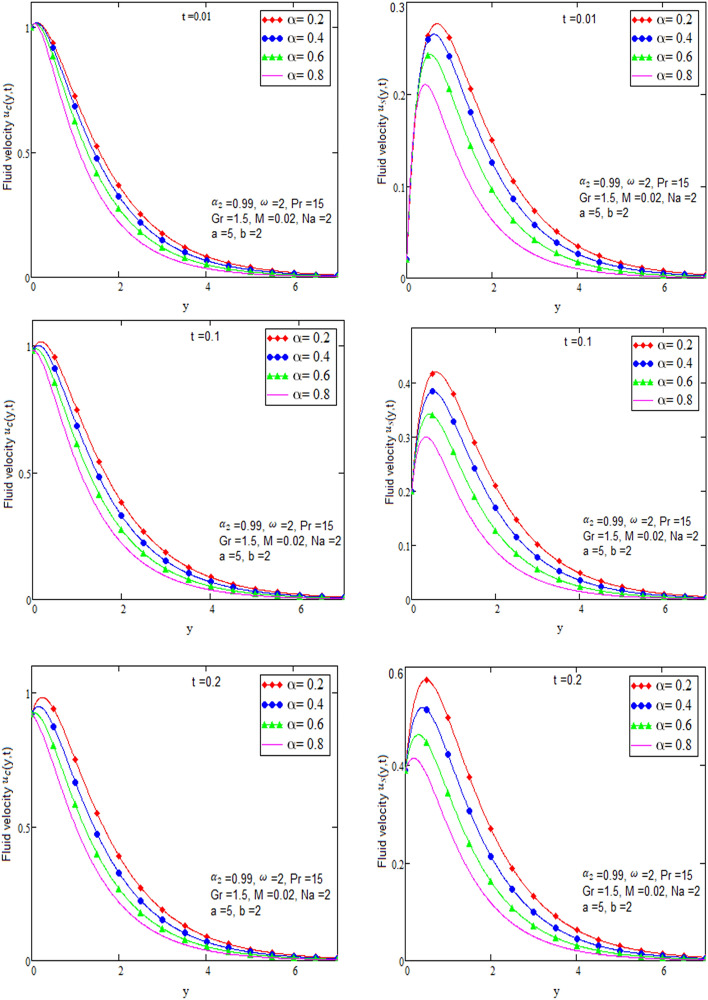
Graph of velocity for different value of alpha α in case of cosine and sine oscillation.

**Figure 6:**
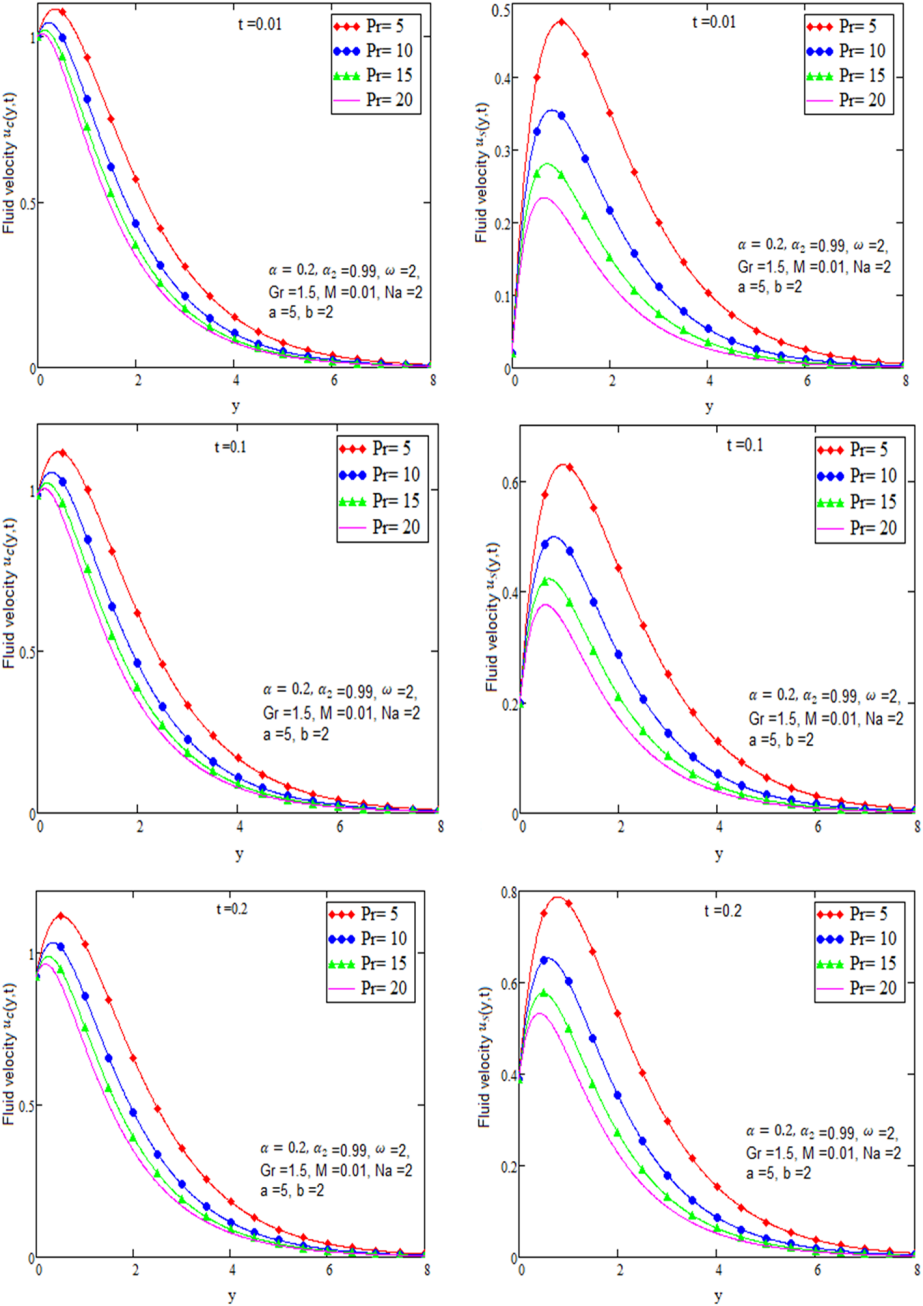
Graph of velocity for different value of Prandtl number Pr in case of cosine and sine oscillation.

**Figure 7:**
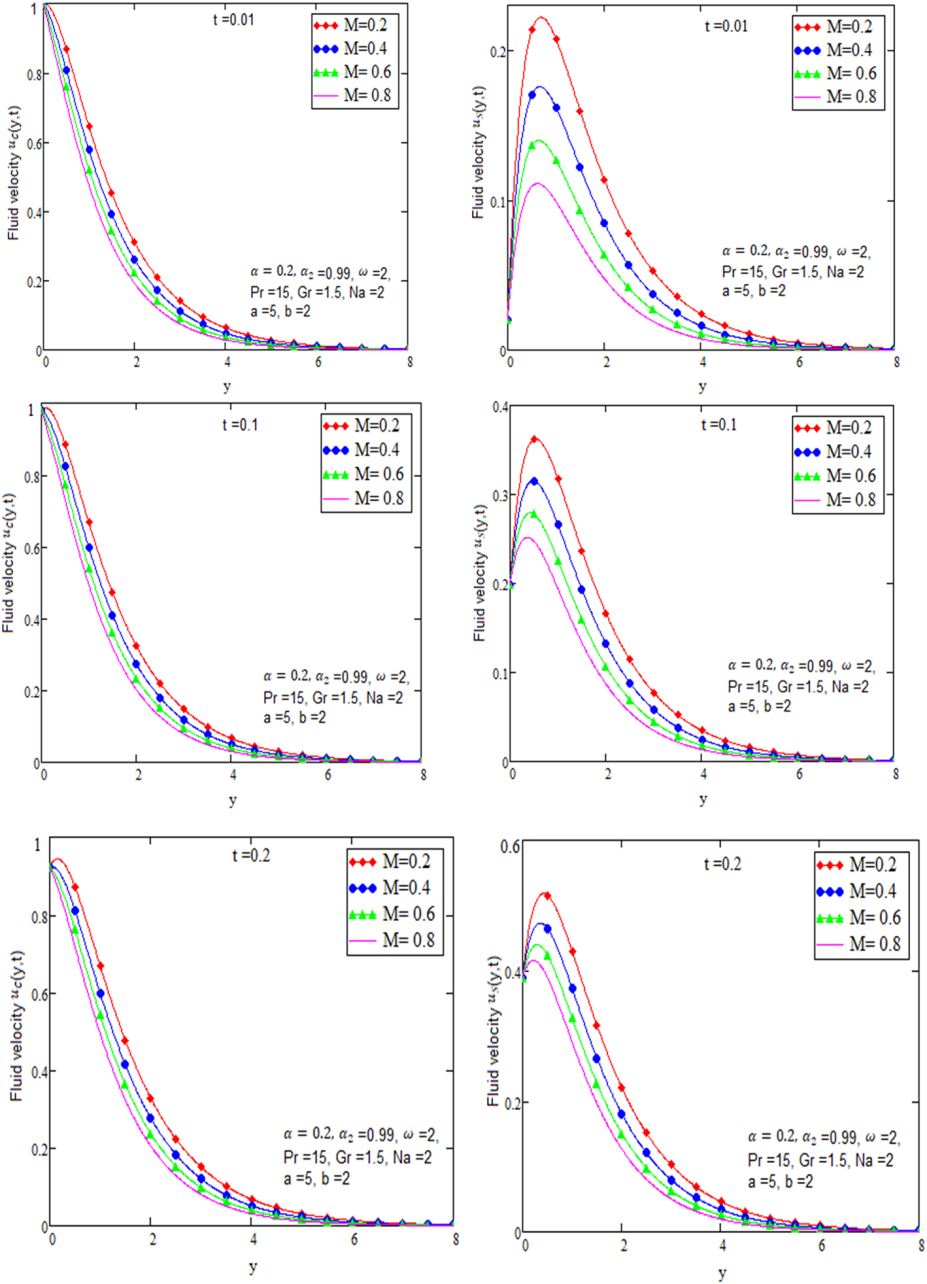
Graph of velocity for different value of M in case of cosine and sine oscillation.

**Figure 8:**
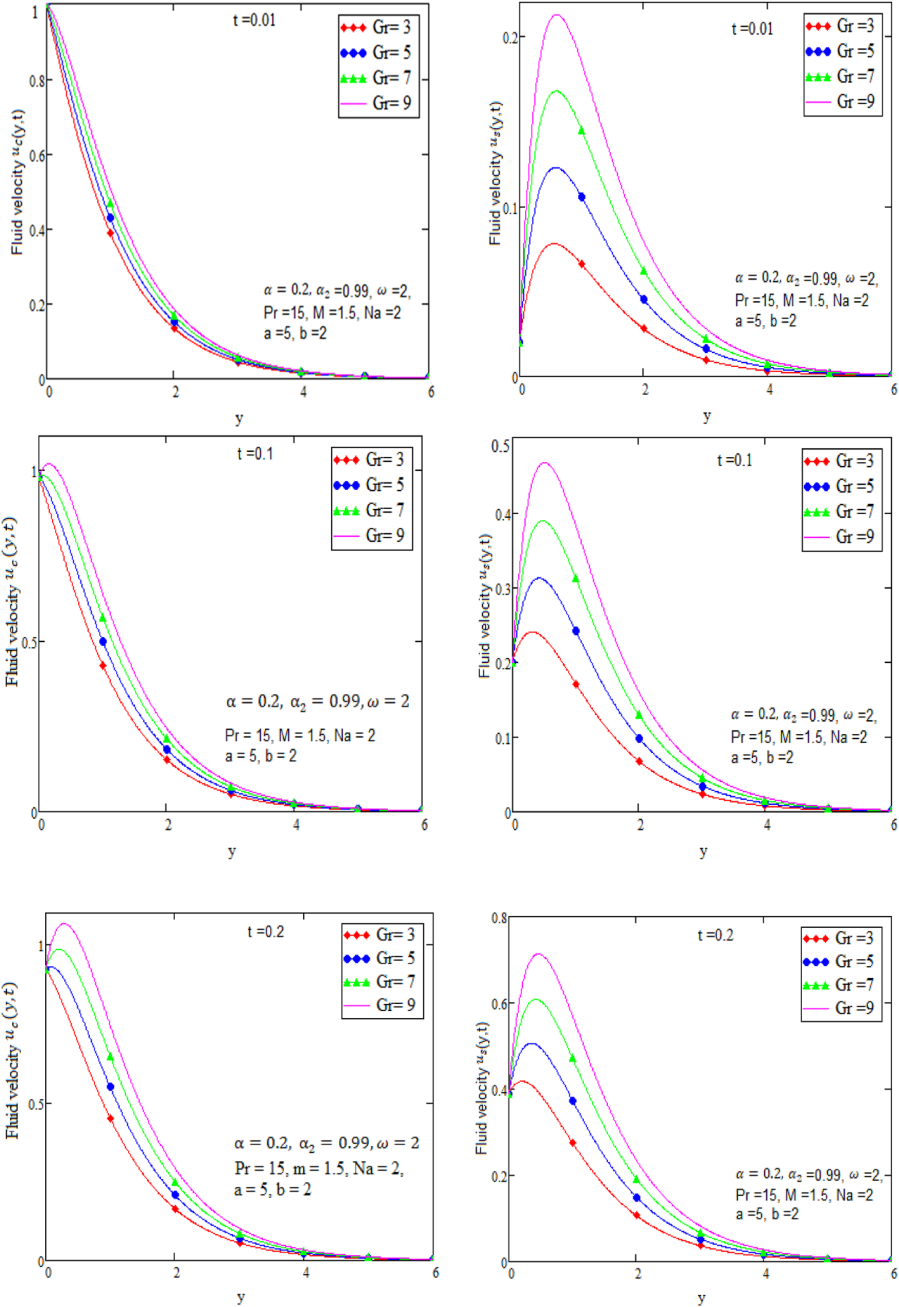
Graph of velocity for different value of Grashof number Gr in case of cosine and sine oscillation.

By using Mathcad software different physical parameters are sketch to analyze the effects of fluid velocity and temperature. The parameter alpha α in Fig. 2, Prandtl number Pr in Fig. 3 and heat radiation in Fig. 4 are drawn for temperature field, while for velocity field alpha α in Fig. 5, Prandtl number Pr in Fig. 6, Magneto hydro dynamic MHD in Fig. 7 and Grashof number Gr in Fig. 8 are presented.

**Figure 9:**
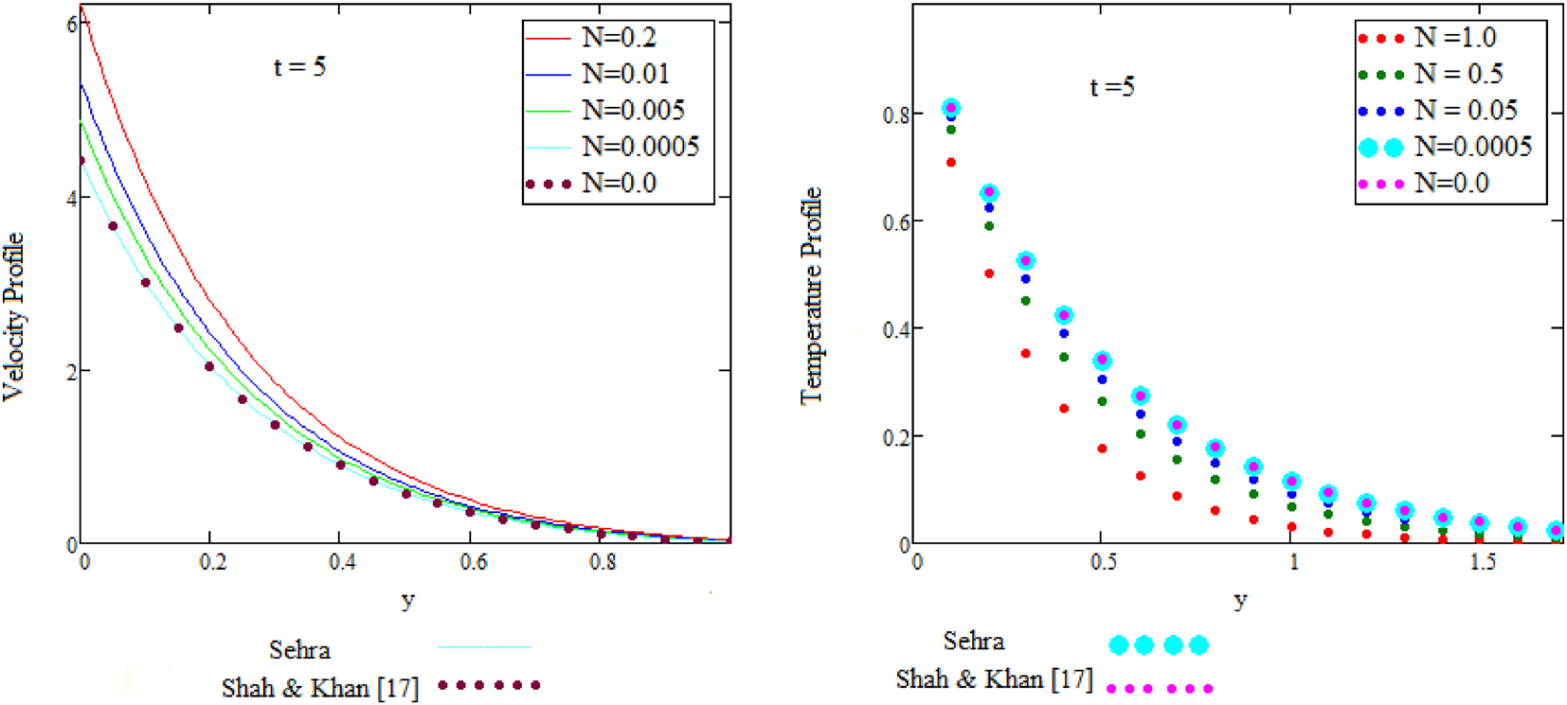
Velocity and temperature profile with comparison of published literature published by Shah & Khan.

Figure 2 is sketch to check the effects of temperature and alpha α in which we saw this temperature is rises by growing the value of α, the boundary layer thermal thickness is rises with parameter alpha α and time t. Figure 3 is sketch to check the influence of temperature and Prandtl Pr in which we observed that the temperature decreases by rising value of Prandtl Pr, the thickness of thermal boundary layer is decreasing with the parameter Prandtl number Pr and time t and diffusivity of temperature is large. Figure 4 is sketch to check the effects of temperature and heat radiation N it has been investigated, by growing the small value of heat radiation N the temperature is also rises. The graph is plotted temperature versus y. Figure 5 is drawn to check the influence of alpha α, both cases of sine and cosine oscillation are discussed in which we investigate this fluid velocity decreases by increasing the value of alpha α. This graph shows both the effects of cosine and sine oscillation for fluid. Effects of sine oscillation are greater than cosine oscillation by increasing the time t. Figure 6 is drawn to examine Prandtl Pr effects over fluid velocity, individually the cases of sine & cosine oscillation were considered in which we emphasized this by growing the small values of Prandtl Pr number, velocity decreases. Figure 7 is drawn to study the behaviour of Magnetohydrodynamic M, both the cases of sine and cosine oscillation are considered in which we noticed by small values of MHD increasing the velocity has decreases. Effects of sine oscillation are greater than cosine oscillation by increasing the time t. Figure 8 is drawn to observe the influence of Grashof number, both cases of sine and cosine oscillation are considered in which we noticed that the fluid velocity is increases by growing the value of Gr. The effects of sine oscillation are greater than cosine oscillation by increasing the time t. In Fig. 9 we compared the obtained solutions as limiting cases with those obtained by Shah and khan^16^.

### Numerical tables

Numerical results of skin-friction and Nusselt number at the plate $\left( {y = 0} \right)$ are presented in Tables 1 and 2 for different values of $\left( t \right)$, $\left( \alpha \right)$, $\left( N \right)$, $\left( M \right)$,$\left( {\Pr } \right)$ and $\left( {Gr} \right)$. It is observed from Table 1 that skin friction $\left( \tau \right)$ increases with an increase in $\left( t \right)$, $\left( N \right)$ and $\left( {Gr} \right)$ while the result is reversed with increase in $\left( \alpha \right)$, $\left( M \right)$ and $\left( {\Pr } \right)$ in Table 1. Numerical results of Nusselt number $\left( {Nu} \right)$ at the plate $\left( {y = 0} \right)$ are expressed in Tables 2 for different values of $\left( t \right)$, $\left( \alpha \right)$, $\left( N \right)$ and $\left( {\Pr } \right)$. Table 2 shows that the Nusselt number Nu which determines the rate of heat transfer at the plate increases as $\left( \alpha \right)$ and $\left( {\Pr } \right)$ progresses while the result is reversed with increase in $\left( t \right)$ and $\left( N \right)$.

**Table 1:** Skin-friction.

$t$	$\alpha$	$N$	$M$	$\Pr$	$Gr$	$\tau$
0.1	0.2	2	0.02	15	1.5	0.419
0.2	0.2	2	0.02	15	1.5	0.568
0.1	0.3	2	0.02	15	1.5	0.38
0.1	0.2	3	0.02	15	1.5	0.457
0.1	0.2	2	0.03	15	1.5	0.404
0.1	0.2	2	0.02	16	1.5	0.397
0.1	0.2	2	0.02	15	1.6	0.496

**Table 2:** Nusselt number.

$t$	$\alpha$	$N$	$\Pr$	$Nu$
0.1	0.2	2	7	1.24
0.2	0.2	2	7	0.126
0.1	0.3	2	7	1.306
0.1	0.2	3	7	1.062
0.1	0.2	2	8	1.326

## Conclusions

Unsteady free convection flow of generalized second grade fluid over an infinite vertical plate is studied. The flow is analyzed under the effect of magneto hydrodynamic and radiation together with heat transfer. Moreover On the thermal aspects of the infinite vertical plate, we are taking into account the exponential heating phenomena. The Caputo–Fabrizio derivative has been applied to the set of dimensionless governing equation. Exact solution of the problem is obtained through Laplace transorm technique.The profiles (temperature and velocity) are analyzed graphically for both sine and cosine oscillations of the plate for distinct physical parameters.

It is observed that.

1. By increasing the fractional parameter α and radiation N, temperature is also increases.
2. With the increases of Prandtl number the temperature can be decreased.
3. Velocity is decreases by increasing α parameter and hence velocity and temperature have opposite behavior for α parameter.
4. With large value of Prandtl number the fluid velocity tends to decreasing.
5. The motion of the fluid is decreasing for growing value of MHD.
6. The velocity is growing by large value of Gr.

## Supplementary Information

Supplementary Information.

## Data Availability

The datasets analyzed during the current study available from the corresponding author on reasonable request.

## List of symbols

C_p_: Specific heat
g: Gravity (acceleration)
N: Parameter of radiation
Gr: Grashof number
Tω: Wall temperature
ν: Viscosity (kinematic)
ρ: Fluid density
σ: Electrical conductivity
T: Fluid temperature
Pr: Prandtl number
k*: Coefficient of mean absorption
σ*: Constant (Stefan–Boltzmann)
α_1_: Grade second parameter
B_0_: Uniform magnetic field
Β_T_: Thermal expansion volumetric coefficient
t: Time
p: Laplace transform parameter
k: Thermal conductivity

## References

[1] Kulish VV, Lage JL (2002). Application of fractional calculus to fluid mechanics. J. Fluids Eng..

[2] Debnath L (2003). Recent applications of fractional calculus to science and engineering. Int. J. Math. Math. Sci..

[3] Hilfer R (2008). Threefold introduction to fractional derivatives. Anomal. Transport Found. Appl..

[4] Gorenflo R, Mainardi F, Moretti D, Paradisi P (2002). Time fractional diffusion: A discrete random walk approach. Nonlin. Dyn..

[5] Caputo M, Fabrizio M (2015). A new definition of fractional derivative without singular kernel. Progr. Fract. Differ. Appl..

[6] Wenchang T, Mingyu X (2004). Unsteady flows of a generalized second grade fluid with the fractional derivative model between two parallel plates. Acta Mech. Sin..

[7] Friedrich CHR (1991). Relaxation and retardation functions of the Maxwell model with fractional derivatives. Rheol. Acta.

[8] Wenchang T, Wenxiao P, Mingyu X (2003). A note on unsteady flows of a viscoelastic fluid with the fractional Maxwell model between two parallel plates. Int. J. Non-Linear Mech..

[9] Hayat T, Nadeem S, Asghar S (2004). Periodic unidirectional flows of a viscoelastic fluid with the fractional Maxwell model. Appl. Math. Comput..

[10] Yin Y, Zhu KQ (2006). Oscillating flow of a viscoelastic fluid in a pipe with the fractional Maxwell model. Appl. Math. Comput..

[11] Sene N (2022). Analytical investigations of the fractional free convection flow of Brinkman type fluid described by the Caputo fractional derivative. Res. Phys..

[12] Yavuz M, Sene N, Yıldız M (2022). Analysis of the influences of parameters in the fractional second-grade fluid dynamics. J. Math..

[13] Jamil M, Rauf A, Zafar AA, Khan NA (2011). New exact analytical solutions for Stokes’ first problem of Maxwell fluid with fractional derivative approach. Comput. Math. Appl..

[14] Khan M, Hayat T, Asghar S (2006). Exact solution for MHD flow of a generalized Oldroyd-B fluid with modified Darcy’s law. Int. J. Eng. Sci..

[15] Khan M, Ali SH, Qi H (2009). On accelerated flows of a viscoelastic fluid with the fractional Burgers’ model. Nonlinear Anal. Real World Appl..

[16] Shah NA, Khan I (2016). Heat transfer analysis in a second grade fluid over and oscillating vertical plate using fractional Caputo–Fabrizio derivatives. Eur. Phys. J. C.

[17] Abbas A, Shafqat R, Jeelani MB, Alharthi NH (2022). Convective heat and mass transfer in third-grade fluid with Darcy-Forchheimer relation in the presence of thermal-diffusion and diffusion-thermo effects over an exponentially inclined stretching sheet surrounded by a porous medium. Adv. CFD Convect. Heat Transf..

[18] Siddique I, Tlili I, Bukhari SM, Mahsud Y (2021). Heat transfer analysis in convective flows of fractional second grade fluids with Caputo–Fabrizio and Atangana-Baleanu derivative subject to Newtonion heating. Mech. Time-Dependent Mater..

[19] Haq SU, Shah SIA, Jan SU, Khan I (2021). MHD flow of generalized second grade fluid with modified Darcy’s law and exponential heating using fractional Caputo–Fabrizio derivatives. Alex. Eng. J..

[20] Saqib M, Ali F, Khan I, Sheikh NA, Jan SAA (2018). Exact solutions for free convection flow of generalized Jeffrey fluid: A Caputo–Fabrizio fractional model. Alex. Eng. J..

[21] Raptis A, Perdikis C, Takhar HS (2004). Effect of thermal radiation on MHD flow. Appl. Math. Comput..

[22] Siddheshwar PG, Mahabaleswar US (2005). Effects of radiation and heat source on MHD flow of a viscoelastic liquid and heat transfer over a stretching sheet. Int. J. Non-Linear Mech..

[23] Hayat T, Qasim M (2011). Radiation and magnetic field effects on the unsteady mixed convection flow of a second grade fluid over a vertical stretching sheet. Int. J. Numer. Methods Fluids.

