# Supplementary material for: Heat transfer of generalized second grade fluid with MHD, radiation and exponential heating using Caputo–Fabrizio fractional derivatives approach
Source: Sci Rep. 2023 Mar 30;13:5220. doi: 10.1038/s41598-022-22665-4 (PMC10063610; doi:10.1038/s41598-022-22665-4)
Supplement: Supplementary file 1 — Supplementary Information. [file 41598_2022_22665_MOESM1_ESM.pdf]

Appendix

$$\Phi\left(y,q;k,l\right)=\frac{1}{q}\left(\exp(-y\sqrt{\frac{kq}{q+l}}\right),$$

$$\varphi\left(y,t;k,l\right)=L^{-1}\left\{\Phi\left(y,q;k,l\right)\right\}=1-\frac{2k}{\pi}\int_0^\infty\frac{\sin\left(yx\right)}{x\left(k+x^2\right)}\exp\left(-\frac{ltx^2}{k+x^2}\right)dx, \qquad \text{(A1)}$$

$$F\left(y,q;k,l,m\right)=\frac{1}{q-m}\exp\left(-y\sqrt{\frac{kq}{q+l}}\right)=\Phi\left(y,q;k,l\right)+\psi\left(y,q;k,l,m\right), \qquad (A2)$$

$$\psi\left(y,q;k,l,m\right)=\frac{1}{q-m}\Phi\left(y,q;k,l\right),$$

$$\psi\left(y,t;k,l,m\right)=L^{-1}\left\{\psi\left(y,q;k,l,m\right)\right\}$$

$$=\exp\left(mt-y\sqrt{\frac{km}{l+m}}\right)-1-\frac{2km}{\pi}\int_0^\infty\frac{\sin\left(yx\right)}{x\left[km+\left(l+m\right)x^2\right]}\exp\left(-\frac{ltx^2}{k+x^2}\right)dx, \qquad \text{(A3)}$$

$$h(\tau)=L^{-1}\left\{\exp\left(-y\sqrt{\frac{a_1p+a_2}{p+a_3}}\right)\right\}$$

$$=\delta\left(t\right)e^{-y\sqrt{a_1}}+\int_0^\infty\frac{y}{2u\sqrt{\tau}}\sqrt{\frac{a_1b_2-b_1}{t}}\times e^{-\frac{y^2}{4u}}\times e^{-b_2t-a_1u}\times l_1\left(2\sqrt{\left(a_1b_2-b_1\right)ut}\right)du. \qquad \text{(A4)}$$
